# Microbiome drives age-dependent shifts in brain transcriptomic programs at the single-cell level in *Drosophila*

**DOI:** 10.1038/s41522-025-00781-z

**Published:** 2025-08-12

**Authors:** Dianshu Zhao, Russel T. Shiga, Zhangrong Song, Runhang Shu, Lipin Loo, Adam Chun Nin Wong

**Affiliations:** 1 Entomology and Nematology Department, Institute of Food and Agricultural Sciences, University of Florida, Gainesville, FL USA; 2 School of Life and Environmental Sciences and the Charles Perkins Centre, The University of Sydney, Sydney, NSW Australia; 3 Genetics Institute, University of Florida, Gainesville, FL USA

**Keywords:** Microbiome, Next-generation sequencing

## Abstract

The gut microbiome plays a critical role in brain function and the brain-gut axis, yet its cellular and molecular mechanisms remain unclear. Here, we present the first comprehensive single-cell transcriptomic atlas of brain cells from adult *Drosophila melanogaster* raised under axenic and microbiome-associated conditions, spanning young and old ages. Profiling 34,427 cells across 101 clusters, we annotated 56 cell types and identified cell type-specific gene signatures influenced by the microbiome. Transcriptional shifts were most pronounced in old flies, with glial cells and dopaminergic neurons among the most microbiome-responsive cell types. Differentially expressed genes (DEGs) were enriched in pathways related to mitochondrial activity, energy metabolism, and Notch signaling. We also quantified age-associated changes in the gut microbiome, observing reduced *Acetobacter* dominance and increased microbial diversity that corresponded with heightened brain transcriptional responses. These findings illuminate the cell type-specific impacts of the microbiome on brain gene expression and lay the groundwork for understanding the molecular underpinnings of the microbiome-gut-brain axis.

## Introduction

Animal organ systems are increasingly recognized to function in the context of host-microbe symbiosis. Notably, the brain–gut axis exemplifies the dynamic bidirectional communication between the central nervous system (CNS) and the gastrointestinal tract, in which modulation by the gut microbiome has emerged as a crucial determinant of wide-ranging neurological and behavioral outcomes. Mechanistically, considerable progress has been made in identifying microbiome-derived molecules and their regulatory roles in brain functions or behavior. The gut microbiome is capable of generating an array of bioactive compounds, such as short-chain fatty acids (SCFAs), biogenic amines, neurotransmitters and their precursors, which shape brain development and neurophysiological processes^1–3^. Gut bacterial components can also interact with host immune and metabolic networks. Examples include bacterial lipopolysaccharides and flagella sensed by specific receptors in enteroendocrine cells, resulting in the secretion of peptide hormones^4^. Moreover, the influence of the gut microbiome extends beyond these molecules, impacting whole-organismal physiology and behavior. Intriguingly, preclinical studies suggest that fecal microbiome transplants from human patients with Parkinson’s disease (PD) can induce similar behavioral symptoms in mice, accompanied by changes in brain gene expression^5^. These findings highlight the causal role of the gut microbiome in brain function and underscore the importance of studying brain gene expression to identify the underlying mechanisms.

The vast diversity of microbial effectors and behaviors modulated by the microbiome suggests that the gut microbiome likely influences brain functions through multiple signaling pathways. However, we know little about which and how brain gene regulatory networks respond to the microbiome. Resolving the brain’s response to the microbiome is an essential first step toward elucidating the brain–gut axis, requiring high cellular resolution due to the brain’s heterogeneous, functionally differentiated cell types. However, the highly complex human brain, comprising over 85 billion neurons and other cells, and a microbiome with 500–1000 taxa, presents significant challenges^6,7^. The experimentally tractable *Drosophila melanogaster*, which has a less diverse microbiome, a brain with ~12,000 neurons, yet exhibits complex behaviors, offers a simpler model to address this important knowledge gap^8,9^.

To probe the brain cells and gene regulatory networks influenced by the gut microbiome, we present here a single-cell RNA sequencing (scRNA-seq) analysis comparing the brains of microbiome-associated and axenic *D. melanogaster* at young and old ages. Given that aging can profoundly impact brain gene expression, neural plasticity, and neuronal structure, while simultaneously inducing shifts in the composition and function of the gut microbiome^10–14^, we hypothesize that the brain–gut axis is modulated dynamically over the lifespan. We included age as a variable in our study design to capture both age-dependent and age-independent microbiome effects on brain responses. Leveraging existing data resources, we developed a comprehensive reference database for *D. melanogaster* brain cell types by integrating data from multiple scRNA-seq studies and introduced an improved workflow for cell clustering and annotation. This allows us to construct a *Drosophila* single-cell transcriptome atlas with 56 confidently annotated cell types. Our analyses suggest that the gut microbiome affects fly brain gene expression in a cell type-specific manner, with more pronounced effects observed in old flies. Notably, glial cells, particularly subperineural glia that is crucial for maintaining the blood-brain barrier, as well as neurotransmitter-producing neurons such as dopaminergic neurons, are among the most microbiome-responsive. We also observed overrepresentation of genes involved in mitochondrial activities and energy metabolism most affected by the microbiome. These findings highlight the crucial role of the gut microbiome in modulating brain cell-specific programs and the dynamic changes with age.

## Results

### Single-cell transcriptome atlas of *Drosophila* brain cells from microbiome-associated and axenic flies

**Fig. 1:**
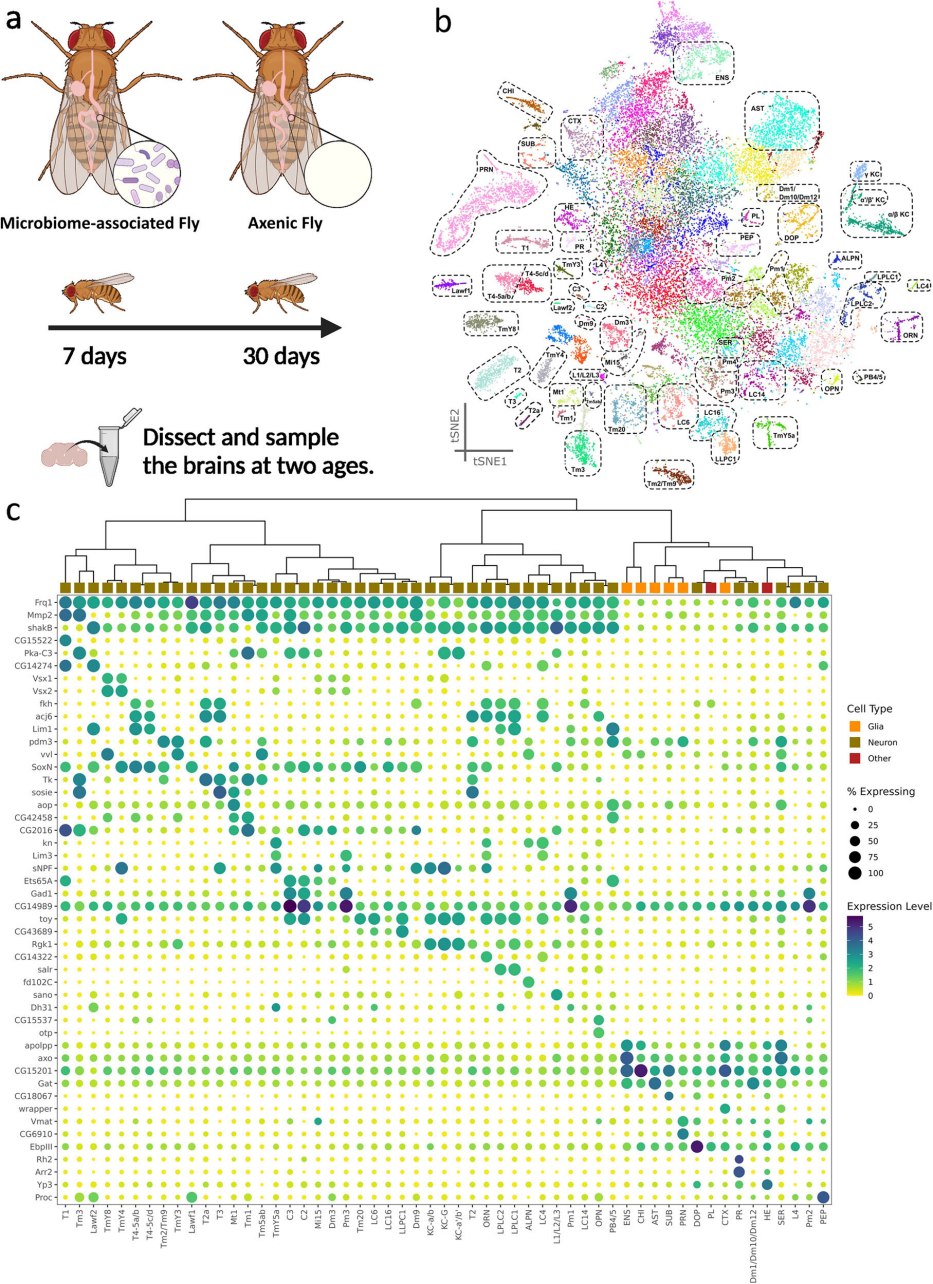
Single-cell transcriptomic profiling of the *Drosophila* brain across different bacterial conditions and ages. **a** Sampling of *Drosophila* brains from adult microbiome-associated and axenic flies (female) at two distinct ages. **b** t-distributed stochastic neighbor embedding (t-SNE) visualization of cells from all treatment groups. **c** Identified cell types from single-cell RNA-seq clustering analysis: (Top) dendrogram illustrating the hierarchical relationships among identified cell types; (Bottom) expression levels and proportions of selected marker genes for identified cell types.

To explore cellular diversity and microbiome-responsive transcriptional signatures in the fly brain, we performed scRNA-seq on cells dissociated from the brains of microbiome-associated and axenic female *D. melanogaster* adults. To account for age effects, we sampled brains from flies of two distinct ages, at 5–7 days (young) and 27–30 days (old) (Fig. 1a). After quality control, we obtained transcriptomes of 34,427 high-quality cells, in which we detected 14,355 protein-coding genes and 3413 non-coding genes. The sequencing depth averaged 1242 transcripts (unique molecular identifiers, UMIs) and 752 genes per cell.

A major challenge in analyzing scRNA-seq data lies in cell identity annotation, particularly in complex brain tissues with diverse cell types lacking well-defined gene markers. To address this, we constructed a local reference database using data from previous scRNA-seq studies on *Drosophila* brain tissues (Materials and Methods). To optimize cell clustering parameters that best delineate the cell types, we used the Leiden algorithm^15^ to calculate cell type clustering results based on two key parameters: the number of principal components (PCs) and resolution. Given the lack of clear guidelines for parameter optimization in the literature, we followed the approach outlined by Özel et al. (2020). We systematically evaluated 88 parameter combinations to identify the optimal settings. We determined that the combination of 45 PCs and a resolution of 8.0 yielded the best alignment with our reference, as determined by Pearson correlation coefficient analysis (Fig. S1). Following parameter optimization, we manually validated the cell clusters, merging those with similar transcriptome profiles into the same cell type (Fig. S2). As a result, we grouped the 34,427 cells into 101 clusters and generated the expression profile for each cluster (Supplementary Data S1).

Among the 101 cell clusters, we successfully identified 56 cell types with known gene expression patterns from published papers (Figs. 1b, c and S1). Our annotated cell types include neurons from the central nervous system and optic lobe, various glial cell types, and other non-neuronal cell types such as hemocytes. In the optic lobe, we identified the centrifugal neurons (C2, C3), distal medulla neurons (Dm1, Dm3, Dm9, Dm10, Dm12), lamina neurons (L1, L2, L3), lamina wide-field neurons (Lawf1, Lawf2), lobula columnar neurons (LC4, LC6, LC14, LC16), lobula-lobula plate columnar neuron (LLPC1), lobula plate-lobula columnar neurons (LPLC1, LPLC2), medulla intrinsic neuron (Mi15), medulla tangential neuron (Mt1), proximal medulla neurons (Pm1, Pm2, Pm3, Pm4), T neurons (T1, T2, T3, T4–5), and transmedullary neurons (Tm1, Tm2/Tm9, Tm3, Tm20, TmY3, TmY5a). In the central nervous system, we detected the Kenyon cells (KC – α/β, KC–α’/β’, KC–γ), olfactory projection neurons, olfactory receptor neurons, and protocerebral bridge neurons (PB4/5).

### DEGs across cell types influenced by the gut microbiome

With a clustered and annotated single-cell transcriptome atlas, we then examined the impact of the gut microbiome on gene expression by identifying differentially expressed genes (DEGs) within each cell type. Given the bimodal expression patterns observed in scRNA-seq data due to inherent cell-to-cell variability^16^, we used the MAST (model-based analysis of single-cell transcriptomics) framework to account for variations in cellular detection rates and gene read counts^17^. We calculated the estimated log fold change (logFC) and false discovery rate (FDR) for each gene, comparing brains from microbiome-associated and axenic flies separately for young and old groups. This analysis identified 9787 DEGs in old flies and 8290 DEGs in young flies. Applying an FDR cutoff of 0.05 significantly reduced the number of DEGs in the young group to 3619 compared to 9674 in the old fly group.

LogFC and FDR are typically evaluated separately in DEG analysis. The correlation between logFC and variance size means that applying a subjective logFC cutoff may omit biologically relevant DEGs. Moreover, considering logFC and FDR separately complicates scRNA-seq data analysis by introducing extra dimensions. To overcome these issues, we used a posteriori information fusion approach proposed by Xiao et al. (2014). This approach integrates logFC and FDR into a single metric, π_g_ (Eq. 1), eliminating the need for cutoffs other than FDR < 0.05.

**Fig. 2:**
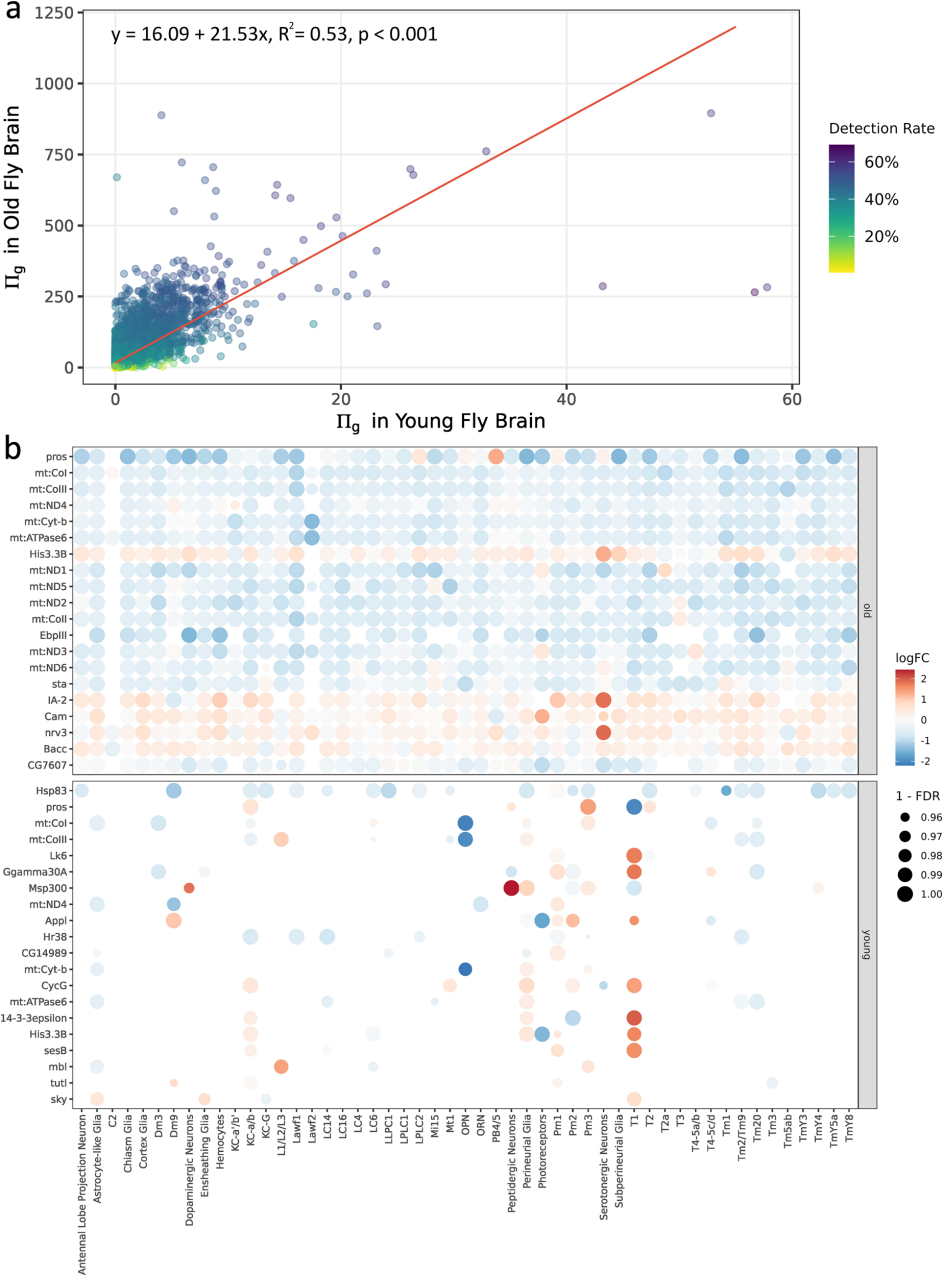
Microbiome-responsive DEGs across brain cell types between young and old flies. **a** The Π_g_ index, calculated for each DEG (microbiome-associated group vs. axenic group) with FDR < 0.05, quantifies the weighted impact of differential expression. Each dot represents a DEG. The color gradient indicates the detection rate, reflecting the proportion of cell types in which a DEG was significantly detected. A linear regression line (solid red) indicates the relationship between Πg in the two age groups. **b** Heatmap of 20 DEGs with the highest Π_g_ values in each age group. Only annotated cell types and protein-coding genes are shown.

After a 0.05 FDR cutoff, we use the ${{\rm{\pi }}}_{g}$ to analyze the distribution of DEGs across different cell types in two age groups. For DEG g detected in n cell types, ${\Pi }_{g}$ (Eq. 2) quantifies its overall presence across multiple cell types and the magnitude of its response to the gut microbiome (Materials and Methods). Our analysis revealed that, as indicated by ${\Pi }_{g}$, the gene expression response to the presence of the gut microbiome is more pronounced in old samples compared to young samples. The average ${\Pi }_{g}$ across all DEGs in old flies is 31.12, whereas in the young flies, it is significantly lower at 0.70. We also applied a linear regression of ${\Pi }_{g}$ of all DEGs between young and old flies. We observed a positive and significant relationship (*R*^2^ = 0.53, *p* < 0.001, Fig. 2a), suggesting that the DEGs we detected are predominantly active in old flies, with relatively lower responses in young fly brains. Interestingly, among the top 20 protein-coding DEGs with highest ${\Pi }_{g}$, genes related to mitochondrial functions are consistently identified across both age groups. In young fly brains, these include mt:CoI, mt:CoIII, mt:ND4, mt:Cyt-b, and mt:ATPase6, while in old fly brains, the list extends to mt:CoI, mt:CoIII, mt:ND1, mt:ND2, mt:ND3, mt:ND4, mt:ND5, mt:ND6, mt:Cyt-b, and mt:ATPase6 (Fig. 2b). Notably, the expression of all these mitochondrial genes is elevated in old axenic fly brains, with most also showing increased expression in the young group (Fig. 2b). In young flies, these DEGs are significant in a limited number of cell types, whereas in old flies, they are significant across most cell types (Fig. 2b). These findings highlight the strong influence of the gut microbiome on mitochondrial activity in the fly brain, especially in old flies.

### Enrichment analysis of the DEGs

**Fig. 3:**
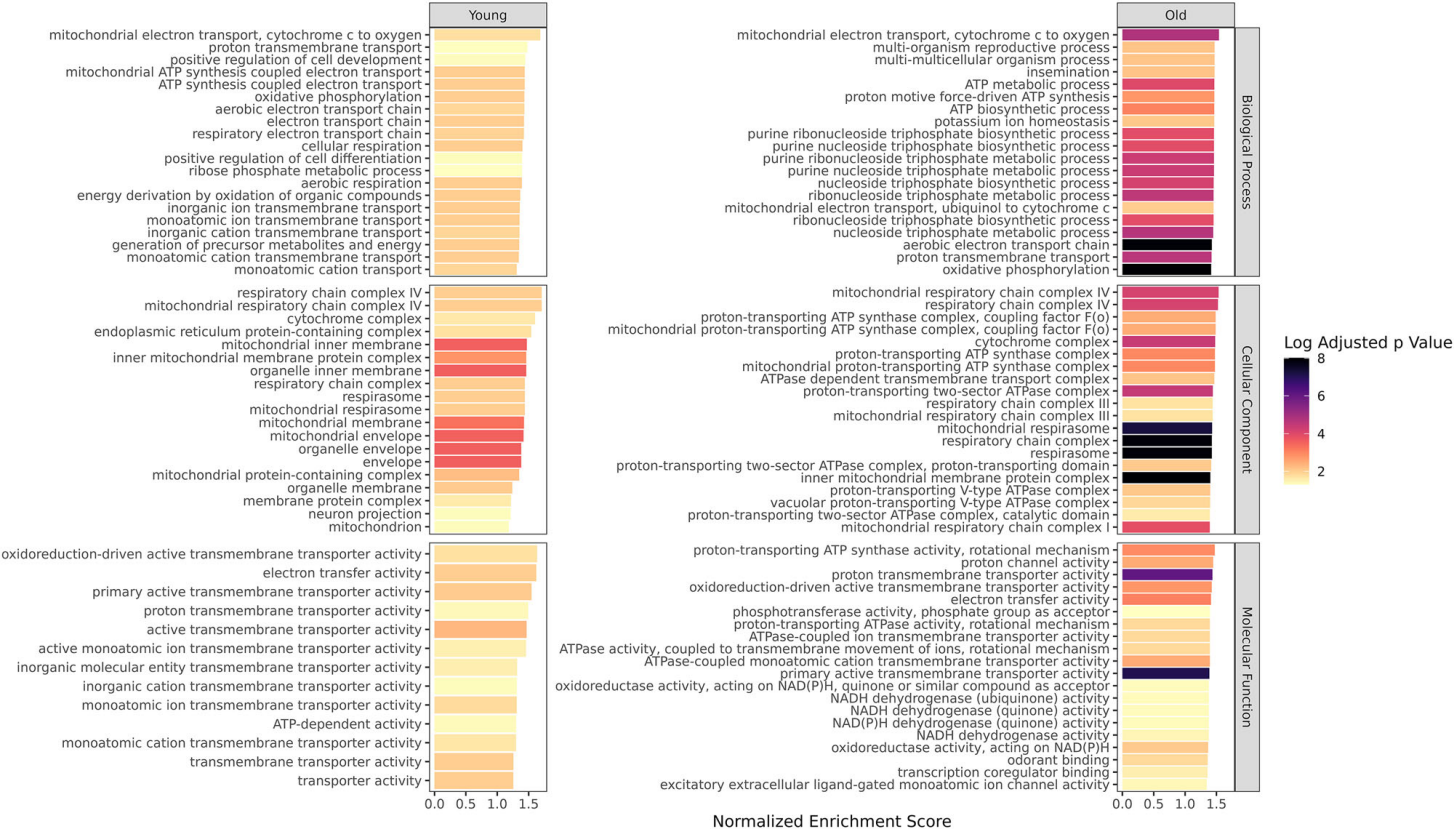
Gene set enrichment analysis of Π_g._ Gene set enrichment analysis (GSEA) was performed using ranked lists of Π_g_ separately for each age group. The top 20 enriched gene sets with the highest normalized enrichment scores (NES) are displayed separately in each domain, along with their corresponding adjusted *p*-values.

To investigate the gene pathways most influenced by the gut microbiome, we conducted Gene Ontology (GO) gene set enrichment analysis by ranking DEGs based on their ${\Pi }_{g}$ values. Our analysis revealed 68 significantly enriched GO terms in the young fly brain influenced by the gut microbiome, compared to 463 in the old fly brain. Several gene sets were notably affected by the gut microbiome in both age groups. Consistent with expectations, the mitochondrial electron transport chain was the most impacted pathway in both young and old fly brains (Fig. 3). Additionally, energy-related pathways, including ATP biosynthetic processes, ATP metabolic processes, and proton transmembrane transport, ranked among the top 10 pathways influenced by the gut microbiome (Fig. 3). Beyond energy production, gene sets associated with behavior and neuron projection were also enriched under the gut microbiome’s influence (Supplementary Data S2).

To better compare the differential effects of the gut microbiome, we applied gene set clustering analysis using a binary cut method. The results indicated substantial overlap in enriched GO terms between young and old fly brains, particularly in clusters related to ion and neurotransmitter transport, mitochondrial and electron transport functions, as well as behavior and development (Fig. S4). However, GO terms grouped into four clusters—transsynaptic signaling, synapse organization and junction, triphosphate biosynthesis, and response to stimuli—were uniquely enriched in the old fly brain under the gut microbiome’s influence (Fig. S4).

### Fly gut microbiome affects brain gene expression in susceptible cell types

Next, we investigated how the fly brain responds to the gut microbiome in a cell type-specific manner. To quantify these responses, we employed two complementary approaches: one based on the global transcriptional profile of each cell type, and another focusing solely on the DEGs at FDR < 0.05.

**Fig. 4:**
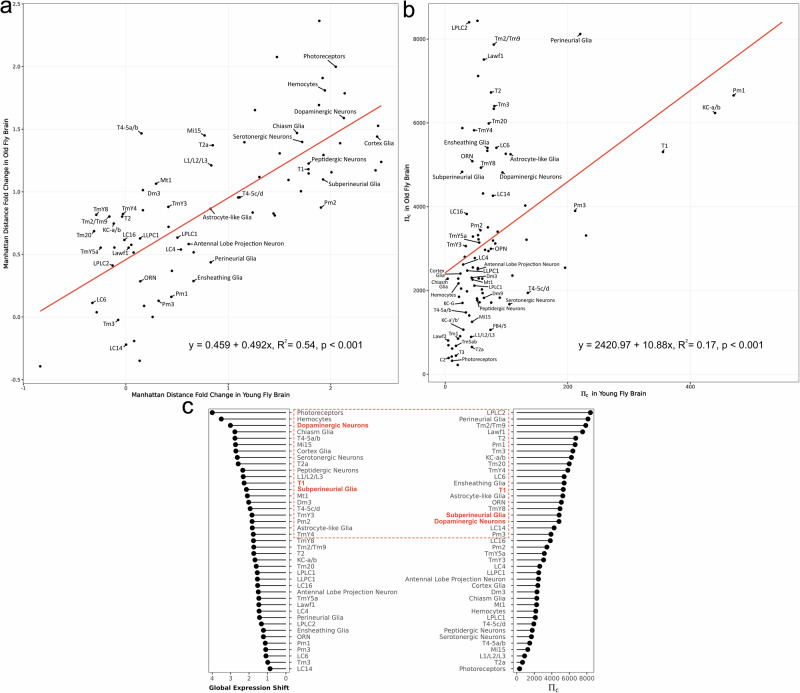
Global and DEG-based transcriptional responses to the gut microbiome across brain cell types. The impact of the gut microbiome on each brain cell type was assessed using two metrics: **a** global transcriptome shift and **b** Πc of all differentially expressed genes (DEGs) with FDR < 0.05. Log-transformed Manhattan distances of gene expression fold changes, and Πc values were calculated separately for microbiome-associated and axenic flies in young and old groups. Each dot represents a cell type. The red regression line indicates the linear relationship between young and old flies, with corresponding statistics shown at the bottom. **c** Rankings of brain cell types in old flies based on global transcriptome shifts and Πc values. The top 20 cell types with the highest values in each metric are circled. Three cell types—T1 neurons, dopaminergic neurons, and subperineurial glia—appear in the top 20 for both metrics and are highlighted in red.

For the global transcriptome shift analysis, each cell type was analyzed independently in the two age groups. We adopted the distance metric method modified from Arneson et al. (2018), substituting Euclidean distance with Manhattan distance to better capture the high-dimensional nature of the dataset^18^. We compared the Manhattan distances of gene expression profiles within each cell type between microbiome-associated and axenic groups (Representative Distance) to determine whether the observed differences exceeded those expected by chance (Null Distance). The influence of the gut microbiome was quantified as the fold change between the Representative Distance and Null Distance (Materials and Methods). Our analysis revealed significant differences in global transcriptome patterns between microbiome-associated and axenic flies in the majority of cell types: among the 101 cell clusters, 74 in the young group and 88 in the old group were significantly influenced by the gut microbiome (Fig. S3). This indicates a pervasive effect of the gut microbiome on brain gene expression in both age groups. Furthermore, we found a positive correlation in global transcriptome shifts between young and old flies (*R*² = 0.54, *p* < 0.001, Fig. 4a), suggesting that the magnitude of transcriptional responses to the microbiome across cell types is consistent between the age groups. To identify the cell types most responsive to the microbiome, we ranked the cell types based on their global transcriptome shift response to the gut microbiome. In young flies, the top five responsive annotated cell types are cortex glia, dopaminergic neurons, photoreceptors, hemocytes, and subperineurial glia. In old flies, the most responsive cell types are photoreceptors, hemocytes, dopaminergic neurons, chiasm glia, and cortex glia.

For the DEG analysis, we quantified microbiome-induced responses in each cell type based on discrete DEGs at FDR < 0.05. and absolute logFC > 0.5. Using ${{\rm{\pi }}}_{g}$ we quantify the magnitude of the response of each cell type to the gut microbiome by aggregating the ${{\rm{\pi }}}_{g}$ values of all DEGs into ${\Pi }_{{\rm{c}}}$ (Eq. 3) within each cell type (Materials and Methods). This analysis showed that the response of different cell types was more pronounced in old flies (average ${\Pi }_{c}$ = 3202.732) compared to young flies (average ${\Pi }_{c}$ = 53.65109, Fig. 4b). Consistent with our previous analysis using ${\Pi }_{g}$, we observed a weak but significant positive correlation in ${\Pi }_{c}$ values across cell types between the two age groups (Fig. 4b, *R*² = 0.17, *p* < 0.001). Both analyses using ${\Pi }_{g}$ and ${\Pi }_{c}$ suggest a more pronounced effect of gut microbiome in old flies. As a result, we focused on the cell types influenced by the gut microbiome in old fly samples for further analysis.

By integrating the results from the global transcriptome shift analysis and the DEG-based analysis, we identified cell types in old fly brains that were most responsive to the gut microbiome. Notably, dopaminergic neurons, subperineurial glia, and T1 neurons were consistently ranked among the top 20 most microbiome-responsive cell types in both analyses (Fig. 4c). We then examined DEGs within these cell types with absolute log fold change (logFC) values greater than 0.5.

In dopaminergic neurons, DEGs were enriched in several functional categories. Genes related to hormone metabolism and transport included *CG10553*, *CG1124*, *CG13360*, *CG5867*, *CG5945*, *CG9512*, *Chd64*, *MSBP*, *spidey*, *Eip93F*, *Jheh1*, and *Idgf4*. Among these, *CG9512*, predicted to encode an ecdysone oxidase, was significantly upregulated in aged conventional fly brains (logFC = 2.04), while *CG1124* (*Jhbp1*), which encodes Juvenile Hormone Binding Protein 1, was significantly downregulated by the gut microbiome (logFC = –1.76). Genes involved in energy production and mitochondrial function included *Acbp2*, *alc*, *Gpdh1*, *CG17646*, *mt:ND1*, *mt:ND2*, *mt:ND3*, and *mt:ND6*, with the mitochondrial genes all showing increased expression in old axenic fly brain. Additional genes were associated with olfactory function and detoxification (*Cat* and *Est-6*) and immune response (*vvl*, *glob1*, *Tsf1*, and *Cyp6d5*). Moreover, members of the cytochrome P450 family, such as *Cyp28a5*, *Cyp4p1*, *Cyp6a2*, *Cyp6a8*, *Cyp6w1*, and *Cyp6d5*, also showed a significant increase in expression under the influence of the gut microbiome (Fig. S5).

In subperineurial glia, DEGs were largely associated with energy production and mitochondrial function. These included *ATPsynB*, *blw*, *CG7630*, *COX4*, *COX7A*, *Cyt-c-p*, *levy*, *mt:CoI*, *mt:CoII*, *mt:ND1*, *mt:ND5*, *ND-MLRQ*, *UQCR-14*, and *UQCR-C2*. Similar to the pattern observed in dopaminergic neurons, all mitochondrial genes were upregulated in old flies lacking a gut microbiome. In contrast, *COX4* and *COX7A* exhibited opposite regulation, with significantly higher expression in microbiome-associated flies. Additionally, several DEGs related to cell-to-cell adhesion and Notch signaling were identified, including *CG17739*, *flw*, *tsr*, *E(spl)mβ-HLH*, *Hipk*, and *lola*. All six of these genes were upregulated in microbiome-associated flies (Fig. S5).

In T1 neurons, DEGs were enriched for neurotransmitter receptors (*nAChRalpha4*, *nAChRalpha5*, *nAChRalpha7*, *Octbeta2R*, *AstC-R1*, *GABA-B-R1*), ion channels (*Hk*, *Mid1*, *para*, *pHCl-1*, *rdgA*, *Rdl*, *Rgk3*, *Sh*, *Ca-alpha1D*, *dysc*, *CG42594*, *cac*), and components of cell–cell adhesion and Notch signaling (*CadN*, *Cals*, *cindr*, *Dscam2*, *Nrg*, *Sema2b*, *ths*, *Eaat1*, *heph*, *wry*). All genes in these categories were upregulated in microbiome-associated flies (Fig. S5).

### Age-associated shifts in gut microbiome composition are accompanied by increased diversity and bacterial load

We hypothesize that age-related changes in gene expression in the *Drosophila* brain may be associated with alterations in gut microbiome composition during aging. To investigate this hypothesis, we conducted a comparative analysis of the gut microbiomes of young and old flies using 16S rRNA gene amplicon sequencing. A total of 1,429,145 reads were generated from 16 samples, of which 1,312,948 reads were retained after quality filtering, resulting in an average of 82,059 reads per sample. These reads were clustered into 474 amplicon sequence variants (ASVs) representing 14 phyla.

**Fig. 5:**
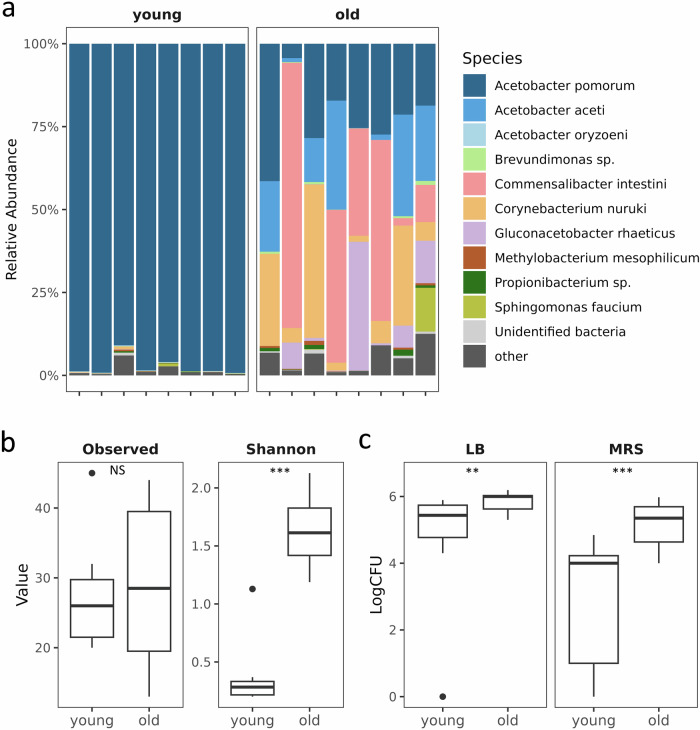
Gut Bacterial community structures in young and old flies. **a** Relative abundance of bacterial species in young and old flies quantified by 16S rRNA gene amplicon sequencing. **b** Comparison of alpha diversity metrics (Observed number of genera and Shannon Index) between the age groups. **c** Quantification of culturable bacteria from the gut on LB and MRS agar plates for young and old flies.

Our findings suggest significant age-associated shifts in both the diversity and abundance of the gut microbiome. Although we did not observe a significant difference in microbial richness between young and old flies (average observed number of genera: young = 27.5, old = 29.0, W = 32.5, *p* > 0.05), the Shannon diversity index showed a significant increase in old flies (average Shannon index: young = 0.38, old = 1.63, W = 61, *p* < 0.001; Fig. 5b), indicating increases in overall diversity driven by evenness with age.

One notable shift was the significant decline in the relative abundance of *Acetobacter pomorum*, dropping from 98% in young flies to 23% in old flies (Fig. 5a). Together with *A. pomorum*, four other taxa *Commensalibacter intestini*, *Acetobacter aceti*, *Corynebacterium nuruki*, and *Gluconacetobacter rhaeticus* collectively accounted for more than 99% of the total reads in both age groups (Fig. 5a). In young flies, the combined relative abundance of these four taxa was below 1%, while in old flies, *C. intestini* reached an average relative abundance of 28.3%, surpassing *A. pomorum*. Additionally, the relative abundances of *C. nuruki*, *A. aceti*, and *G. rhaeticus* increased to 15.6%, 15.5%, and 8.5%, respectively, in the old fly guts (Fig. 5a).

To complement our microbiome sequencing analysis, we performed a colony-forming unit (CFU) assay to quantify the gut microbial loads of both young and old flies using the LB and MRS media. Our CFU data revealed a significant increase in bacterial loads in old flies. Specifically, the average logCFU per fly on LB plates increased from 4.82 in young flies to 5.84 in old flies (*W* = 82, *p* < 0.05). Similarly, on MRS plates, the average logCFU rose from 2.98 in young flies to 5.16 in old flies (*W* = 90.5, *p* < 0.001). These results indicate a substantial increase in microbial load with age (Fig. 5c).

## Discussion

The present study is the first to examine gene expression differences between microbiome-associated and axenic *Drosophila melanogaster* across brain cell populations. Using scRNA-seq, we generated a comprehensive atlas of the adult female brain and revealed microbiome-dependent transcriptional changes. The dataset includes 34,427 high-quality cells across 101 clusters from both young and old groups. Compared to previous *Drosophila* brain scRNA-seq studies that identified 39–48 cell types^19,20,21^, our annotation of 56 distinct cell types represents a notable advance in resolution. By integrating transcriptome-wide profiles rather than relying solely on marker gene approaches, we provide a novel and scalable solution for cell type identification in large, complex datasets. Our analyses reveal a pervasive, cell type-specific influence of the gut microbiome on brain gene expression that intensified with age. Cell type-specific responses, such as pronounced transcriptional shifts in the dopaminergic neurons and glia, demonstrate that the microbiome exerts differential effects across brain cell populations. The strong enrichment of microbiome-responsive DEGs involved in mitochondrial activity and energy metabolism is particularly striking. Given the central role of mitochondria in brain energy metabolism and considering that mitochondrial dysfunction and oxidative stress are hallmarks of neurodegenerative diseases such as Alzheimer’s and Parkinson’s, our findings imply a connection between the gut microbiome and mitochondrial homeostasis in the aging brain^22–24^.

We demonstrated that gene expression in glial cells, particularly subperineurial glia, is strongly influenced by the gut microbiome. Subperineurial glia, along with perineurial glia, form the blood-brain barrier (BBB) in the adult brain^25^. The BBB plays a crucial role in brain physiology by regulating molecular exchange between brain tissue and hemolymph^26^. Its activity is closely tied to various aspects of fly physiology, including metabolism, aging, and circadian rhythms^27,28^. Our analysis indicates that the gut microbiome directly impacts the gene expression of glial cells forming the BBB. In subperineurial glia, genes associated with mitochondrial function, Notch signaling, and cell–cell adhesion are upregulated in flies with a regular gut microbiome. This suggests that the gut microbiome may affect the functionality and integrity of the BBB. Supporting this, studies in mammalian models have shown that gut microbiome-derived metabolites can affect BBB integrity and brain health^29,30^. However, direct effects of the gut microbiome on the BBB in *Drosophila* remain to be established. Interestingly, the most pronounced changes in gene expression in glial cells were observed in old flies. Aging in flies is known to involve intestinal barrier dysfunction, potentially allowing leakage of the gut microbiome into the hemolymph. Our results may reflect a direct interaction between the BBB and bacterial cells in the hemolymph that have leaked from the gut, further highlighting the interplay between gut microbiome dynamics and brain physiology.

Our research highlights the value of *D. melanogaster* as a powerful model for microbiome research. With its amenable microbiome and robust genetic tools, *Drosophila* has emerged as a key organism for uncovering the microbiome’s roles in modulating development, nutrition, immunity, and behavior^31–33^. The gut microbiome has been shown to influence a range of behaviors, including sleep, locomotion, foraging, and feeding^34–37^. While these phenotypic effects are well-documented, the underlying host genetic and molecular mechanisms remain poorly understood. By identifying cell type-specific transcriptional programs shaped by the microbiome in the fly, our study lays a foundation for future research into how host-microbe interactions govern complex behaviors and the gut-brain axis.

Finally, the findings also contribute to a broader understanding of microbiome-host interactions in the context of aging. Age-related shifts in the microbiome, including the reduced dominance of bacteria like *Acetobacter* and increased diversity driven by opportunistic taxa, point to a progressive destabilization of the gut ecosystem. These changes are accompanied by increased microbial loads and transcriptional shifts in mitochondrial and ribosomal pathways in the brain, demonstrating how age-related gut dysbiosis can exert systemic effects on other organs. The molecular signatures identified in our study highlight potential pathways through which the microbiome may influence brain metabolism and function, providing a foundation for future investigations into their roles in aging and disease.

While our single-cell atlas of 34,427 adult *Drosophila* brain cells offers the first high-resolution glimpse of how an intact microbiome versus axenic conditions might shape cell-type-specific transcriptional programs in young and old flies, it reflects several practical limitations, some of which are common to single-cell and model organism studies. The shallow sequencing depth typical of scRNA-seq limited the detection of rare cell types, including those in the optic lobes and clock neurons. Pooling of 20 brains per sample was a practical solution to capture sufficient cells for robust clustering and comprehensive gene expression profiling, but precluded analysis of individual-level variability. Our microbiome reconstitution approach using whole-fly homogenate captures complex, age-related gut community shifts, as revealed by our 16S rRNA amplicon sequencing, but it cannot resolve taxon- or strain-specific effects. Future studies using gnotobiotic models will help pinpoint how specific microbial taxa influence brain gene expression to complement our community-level insights. It is also important to note that the *Drosophila* gut microbiome is variable, shaped by interacting factors including host genetics, diet, habitat, and environmental microbial exposure, all of which can influence transcriptional outcomes^38,39^. Testing different genotypes and diets will reveal how genetic background and nutritional condition modulate these effects. Focusing on females, we have yet to explore potential sex-specific transcriptional differences, particularly in pathways related to mitochondrial activity and metabolism. Future research could also leverage single-nucleus RNA sequencing (snRNA-seq) for deeper coverage and improved cell detection, as well as proteomics to link transcriptional and protein changes for more functional insights.

## Methods

### Drosophila husbandry

*D. melanogaster* strain Canton-S was used in this study. Flies were reared on yeast-sucrose media that contained 50 g sucrose, 100 g Brewer’s yeast, and 12 g agar per liter, with 0.04% phosphoric acid and 0.42% propionic acid. They were kept at 25 °C on a 12 h light/dark cycle.

### Fly microbiome treatments

To generate axenic flies, eggs were treated with bleach as described previously^40^, with a few modifications. Briefly, <12-hour-old eggs were collected and dechorionated with 1% sodium hypochlorite (12.5% v/v regular bleach) for 2 min and rinsed in DI water for 3 mins three times. Axenic eggs were then transferred to autoclaved fly food in a laminar flow cabinet, with 20–30 eggs placed in each vial containing 15 mL of fly diet. The axenic status of the resulting adults was confirmed by plating the larval homogenate on LB and MRS agar. To generate microbiome-associated flies, five 1-week-old females were collected from the colony and homogenized in 400 µL phosphate-buffered saline (PBS), then diluted 1:1000. A 50 µL aliquot of the diluted homogenate (0.2% of the food volume and with ~90–120 CFUs) was added to each vial. The adults emerging from these treated eggs were used for the study.

### Preparation of *Drosophila* brain samples

For each condition (young-microbiome-associated, young-axenic, old-microbiome-associated, old-axenic), a pooled sample of 20 brains was collected from eight replicate vials (32 vials total). Brain dissection and tissue dissociation were conducted from 7 AM to 11 AM. Fly brains were dissected in ice-cold Schneider’s *Drosophila* medium (Gibco), with 20 brains pooled for each sample. The dissected brains were immediately incubated with 1.2 mg/mL dispase (Sigma-Aldrich) and 0.6 mg/mL collagenase (Invitrogen) in a shaking incubator (Eppendorf) at 25 °C and 500 rpm for 75 minutes. Following the incubation, the samples were washed three times with ice-cold Dulbecco’s phosphate-buffered saline (DPBS, Gibco) and resuspended in 50 µl DPBS with 0.04% bovine serum albumin (BSA, Sigma). The cell suspension was then passed through a 35 µm cell strainer (Falcon) to remove debris. Cell number and viability were assessed simultaneously using an epifluorescence microscope (Leica) and an Automated Cell Counter to ensure the cell viability exceeded 90%.

### Single-cell library preparation and sequencing

Single-cell libraries were prepared using the Chromium Single Cell 3’ Reagent Kit v2 (10x Genomics) in accordance with the manufacturer’s manual. We conducted droplet-based purification, amplification, and barcoding of single-cell transcriptomes. Briefly, 12,000 cells were added to each channel, estimating recovery of 8000 cells. Nanoliter-scale gel bead-in-emulsions (GEMs) were generated and then reverse transcribed using MasterCycler Pro (Eppendorf) at 53 °C for 45 min and then 85 °C for 5 min. Single-cell droplets were broken, and cDNA was isolated and cleaned using Cleanup Mix containing DynaBeads (Thermo Fisher Scientific). Cleaned cDNA was then amplified using MasterCycler Pro at 98 °C for 3 min, 12 cycles of (98 °C for 15 s, 67 °C for 20 s, 72 °C for 1 min), and 72 °C for 1 min. Amplified cDNA was further fragmented, end-repaired, A-tailed, and index-adaptor ligated using SPRIselect Reagent Kit (Beckman Coulter). Post-ligation product was amplified using MasterCycler Pro at 98 °C for 3 min, 14 cycles of (98 °C for 20 s, 67 °C for 20 s, 72 °C for 1 min), and 72 °C 1 min. Sequencing was performed with a 150pb pair-end kit using the NovaSeq 6000 platform (Illumina) at the University of Florida Interdisciplinary Center for Biotechnology Research (ICBR).

We aligned the sequencing results to the *D. melanogaster* genome assembly BDGP6.48 using 10x Genomics Cell Ranger 7.1.0. Subsequently, we retained the cell barcodes associated with RNA feature numbers falling within the range of 200 to 2500. In addition, cells expressing >20% mitochondrial genes were considered cells with low viability and filtered out from downstream analyses. This threshold was chosen based on standards established in previous single-cell RNA-seq studies, where high mitochondrial content is commonly associated with cellular stress or compromised integrity^41–43^.

### Cell clustering and annotation

We first addressed batch effects from the two sequencing runs using Harmony^44^ with the default settings. We then combined the sequencing data from each treatment group and applied Seurat v5.0^45^ to perform clustering on integrated single-cell transcriptomes with comparable gene transcription patterns. The functions FindNeighbours and FindClusters were utilized with default settings, except for the number of PCs (Harmony adjusted) and resolution parameters. To fine-tune the parameter settings in the calculation, a grid search involving 88 combinations of these parameters was conducted (Fig. S6, No. of PCs from 15 to 50 in steps of 5 and resolution from 1 to 9 in steps of 1). For each combination, we assessed the biological relevance of resulting clusters to known *D. melanogaster* brain cells, to help identify the optimal clustering parameters for our analysis.

We constructed a local reference database containing marker genes for 72 D. melanogaster cell types based on three published single-cell RNA sequencing studies (Supplementary Data S3^19^^,20^^,21^). To assess biological relevance, we employed the ‘Pearson Correlation Gap’ method described by Özel et al. (2021). For each parameter combination, the FindAllMarkers function in Seurat v5.0 was used with a two-sided Wilcoxon rank-sum test to identify positive markers. To establish associations, we calculated the Pearson correlation coefficient between gene markers of each cluster and cell type markers in the local reference database. Clusters were then ranked based on decreasing Pearson correlation coefficient values. For each of the top six best-correlated clusters, we examined their differences in Pearson correlation coefficient values. If one of these differences exceeded 0.15, the cell type was considered to have a clearly matched cluster. This process was repeated for each cluster, each cell type in the local reference database, and each clustering parameter combination, as shown in Fig. S1.

As noted in the previous study^19^, augmenting the number of PCs initially boosts the identification of clear-matched cell types by incorporating more biologically significant components. However, this effect plateaus after a certain threshold, and further increases in PCs would lead to a decrease in the number of clear-matched cell types. A similar trend is observed with the resolution parameter. This pattern has been observed in our analysis and shown in Fig. S1. As a result, we chose 45 PCs and a resolution of 8.0 in the final clustering process that provided a balance between maximizing both the biological relevance and the number of cell types matched, resulting in a total of 114 clusters in our combined dataset across all treatment groups. Of these 114 clusters, 71 were directly identified using the local reference database and merged if identified as the same cell type. In addition, the gene expression patterns of all clusters were manually curated based on their similarity of gene expression pattern, in which 18 clusters were further merged into eight cell types. Our analysis resulted in 101 final clusters (Fig. S2).

The relationships between cell clusters were constructed by first computing UMAP embeddings of single-cell transcriptomic profiles of each cell cluster using Seurat. For each cell cluster, the average UMAP coordinates were calculated, and pairwise Euclidean distances between these centroids were used to perform hierarchical clustering using Ward’s method. The resulting tree was visualized as dendrograms using ggtree 3.14.0^46^.

### Differential gene expression and gene enrichment analyses

For differential gene expression analysis, cell clusters with a minimum of six cells in all four treatment groups were considered (76 out of 101 cell clusters). The gene expression data for each cell cluster were fitted into a hurdle model using MAST 1.28.0. The model coefficient was computed using the model formula: gene transcripts per million ~ fly age + microbial condition + fly age: microbial condition. The model coefficient was further used to determine the fold change of gene expression by comparing the microbiome-associated fly brain to the axenic fly brain in each age group. A likelihood ratio test was then applied to calculate the FDR of each gene within each cell cluster. An FDR < 0.05 was applied to select DEGs for the following analysis. For each DEG *g*, an a posteriori information fusion approach was used to evaluate the logFC and FDR at the same time by calculating ${{\rm{\pi }}}_{g}$ index^47^.

1 $${{\rm{\pi }}}_{g}=\,{\log }_{2}{FC}\,\times \,{(-\mathrm{log}}_{10}{FDR})$$

The magnitude of the effect of gut microbiome to a DEG *g* detected in *n* cell types is then calculated as ${\Pi }_{g}$ in each age group.

2 $${\Pi }_{g}=\,\mathop{\sum }\limits_{1}^{n}\left|{{\rm{\pi }}}_{g}\right|$$

The magnitude of the effect of gut microbiome, in terms of DEG, to a certain cell type *c* with *n* DEG detected is calculated as ${\Pi }_{c}$ in each age group.

3 $${\Pi }_{c}=\,\mathop{\sum }\limits_{1}^{n}\left|{{\rm{\pi }}}_{g}\right|$$

Separate linear regression models were fitted on ${\Pi }_{c}$ and ${\Pi }_{g}$ respectively.

Gene set enrichment analysis (GSEA) was applied to investigate the pathways influenced by gut microbiome in both an overall view and in selected cell types, separately for each age group, using clusterProfiler 4.12.6^48,49^. ${\Pi }_{g}$ was used to rank the DEGs to investigate the influence of gut microbiome across all cell types. ${{\rm{\pi }}}_{g}$ was used to rank the DEGs to investigate the influence of the gut microbiome in selected cell types. The GSEA results were then simplified using rrvgo 1.16.0 with default settings^50^. SimplifyEnrichment 3.20 was used to compare the overlap of the gene sets influenced by the gut microbiome between fly brains from the two age groups^51^.

### Quantitative assessment of global transcriptome shifts

To quantify the effect of gut microbiome on fly brain gene expression at the global level without statistical modeling, we adopted a method first described by Arneson et al.^52^. In the two distinct age groups, we generated two representative cells to simplify the gene expression pattern of each cell type: one representing the microbiome-associated fly brain and the other from the axenic fly brain, by calculating average gene expression (normalized and log transformed) of each gene within each cell type. Subsequently, we calculated the Manhattan distance in gene expression between these two representative cells as a metric to quantify the effect of gut microbiome on each cell type. To determine if the observed Manhattan distance between microbiome-associated and axenic fly brain cells within each cell type reflects the effect was due to the gut microbiome or observed by chance, we estimated a null distribution by calculating the Manhattan distance between randomly sampled cells of the given cell type. This permutation approach was repeated 20,000 times to generate the null distribution, which was compared with the Manhattan distance generated from representative cells in microbiome-associated and axenic groups to determine an empirical *p* value. To correct for multiple comparisons across all the cell types tested, we applied a Benjamini–Hochberg correction to obtain adjusted *p*-values. The intensity of global transcriptome shifts in each cell type was then quantified as log_2_(Manhattan distance between representative cells ÷ average Manhattan distance between randomly sampled cells).

### High-throughput sequencing of the 16S rRNA gene amplicons

5–7-day-old and 27–30-day-old microbiome-associated *Drosophila* females were collected. The insects were surface sterilized by rinsing twice in 0.5% (vol/vol) sodium hypochlorite solution for 1 min, followed by three rinses in sterile water for 1 min each. Guts were then dissected, and eight replicate samples were prepared per age group, each containing a single gut. Total genomic DNA was isolated using a DNeasy blood and tissue kit (Qiagen, Hilden, Germany) following the manufacturer’s instructions. The hypervariable regions (V3–V4) of the bacterial 16S rRNA gene were chosen for amplicon sequencing. The DNA samples underwent library preparation and were sequenced on an Illumina NovaSeq 6000 machine (Illumina, USA) with 2 × 250-bp paired-end (PE) chemistry by Novogene Corporation Inc.

### Amplicon sequence analysis

Pair-end reads were first processed using FastQC 0.11.9. The reads were then imported to the QIIME2 environment. After quality control, reads were denoised and dereplicated, and the chimeras were removed.Amplicon sequence variants (ASVs) were generated using the Divisive Amplicon Denoising Algorithm 2^53^. Taxonomic assignment of ASVs was performed using the q2-feature-classifier with the GreenGene 13.08 database with a 99% identity. Alpha diversity indexes (Observed number of genera and Shannon Index) were calculated using phyloseq 3.20^54^.

### CFU assay

The abundance of cultured bacteria in young (1-week-old) and old (4-week-old) *Drosophila* was quantified. Briefly, individual flies were surface-sterilized by rinsing twice in 0.5% sodium hypochlorite solution (v/v) for 1 minute, followed by three rinses in sterile water for 1 minute each. Surface-sterilized females were homogenized in 500 µl PBS individually using plastic grinding pestles (Capitol Scientific, USA), then serially diluted for plating on LB agar and MRS agar. Colony-forming units (CFUs) was counted after 24 h for LB agar and 48 h for MRS agar. 8 females were used for each age group.

### Statistical analysis

All the statistical analysis is conducted in R v4.3. For alpha diversity indexes and CFU numbers, pairwise comparisons were performed using the Wilcoxon rank-sum test. Statistically significant differences were determined as *P < 0.05, **P < 0.01, ***P < 0.001, and ****P < 0.0001.

## Supplementary information

Supplementary_Figure_and_Data_titles

Supplementary_Data_S1

Supplementary_Data_S2

Supplementary_Data_S3

## Data Availability

Sequencing data supporting this study are openly available as of the date of publication from Gene Expression Omnibus at GSE274419 (single-cell RNA sequencing) and GeneBank PQ775906-PQ776214 (16S rRNA gene amplicon sequencing). All data needed to evaluate the conclusions in the paper are present in the paper and/or the Supplementary Materials.

## Code availability

This paper does not report any original code. Any additional information required to reanalyze the data reported in this paper is available from the lead contact upon request.
