# Supplementary material for: Microbiome drives age-dependent shifts in brain transcriptomic programs at the single-cell level in Drosophila
Source: NPJ Biofilms Microbiomes. 2025 Aug 12;11:162. doi: 10.1038/s41522-025-00781-z (PMC12344059; doi:10.1038/s41522-025-00781-z)
Supplement: Supplementary file 1 — Supplementary_Figure_and_Data_titles [file 41522_2025_781_MOESM1_ESM.pdf]

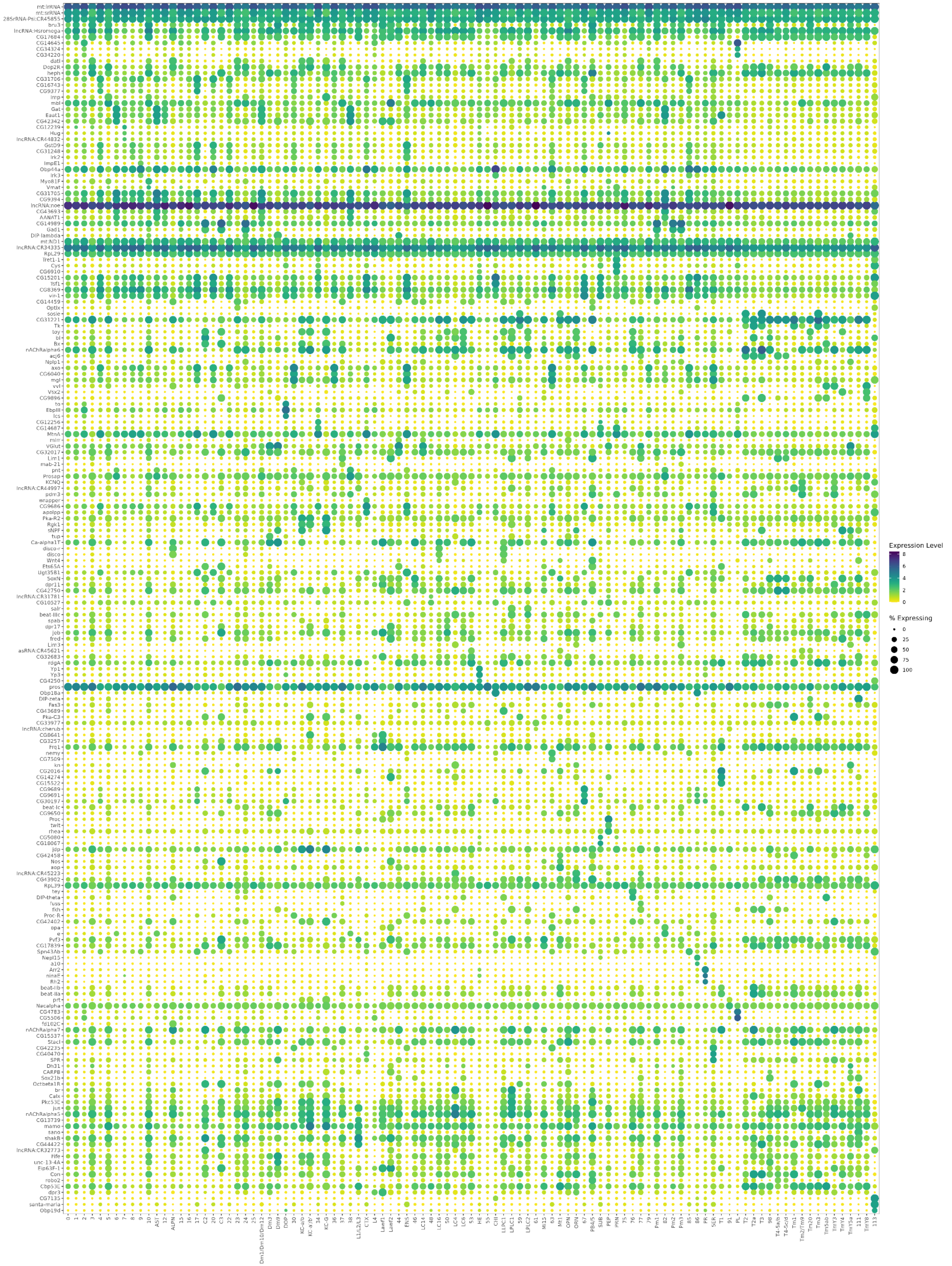
mt:lrRNA
mt:srRNA
28SrRNA-Psi:CR45855
bru3
lncRNA:Hsromega
CG17684
CG14645
CG34324
CG34220
datl
Dop2R
heph
CG31706
CG16743
CG9377
Imp
mbl
Gat
Eaat1
CG42342
CG12239
Hug
lncRNA:CR44832
GstD9
CG31248
Irk2
ImpE1
Obp44a
Irk3
Myo81F
Vmat
CG31705
CG9394
lncRNA:noe
CG43693
AANAT1
CG14989
Gad1
DIP-lambda
mt:ND1
lncRNA:CR34335
RpL29
Iret1-L
Cys
CG6910
CG15201
Tsf1
CG8369
vir-1
CG14459
Optix
sosie
CG31221
Tk
toy
bl
Bx
nAChRalpha6
acj6
Nplp1
axo
CG6040
mgl
vvl
Vsx2
CG9896
to
EbpIII
Ics
CG12256
CG14687
MtnA
mir
VGlut
CG32017
Lim1
mab-21
pnt
Prosap
KCNQ
lncRNA:CR44997
pdm3
wrapper
CG9686
apolpp
Pka-R2
Rgk1
sNPF
tup
Ca-alpha1T
disco-r
disco
Wnt4
Ets65A
Ugt35B1
SoxN
dpr11
CG42750
lncRNA:CR31781
CG10527
salr
beat-IIIc
spab
dpr17
job
fred
Lim3
asRNA:CR45621
CG32683
rdgA
Yp1
Yp3
CG4250
pros
Obp18a
DIP-zeta
Fas3
CG43689
Pka-C3
CG33977
lncRNA:cherub
CG8641
CG3257
Frq1
nemy
CG7509
kn
CG2016
CG14274
CG15522
CG9689
CG9691
CG30197
beat-Ic
CG9650
Proc
twit
rhea
CG5080
CG18067
jdp
CG42458
Nos
aop
lncRNA:CR45223
CG43902
RpL39
tey
DIP-theta
fuss
fkh
Proc-R
CG42402
opa
e
Pvf3
CG17839
Spn43Ab
Nepl15
a10
Arr2
ninaE
Rh2
beat-IIb
beat-IIa
prt
Nacalpha
CG4783
CG5506
fd102C
nAChRalpha7
CG15537
Stacl
CG42235
CG40470
SPR
Dh31
CARPB
Sox21b
Octbeta1R
br
Calx
Pkc53E
jus
nAChRalpha5
CG13739
mamo
sano
shakB
CG44422
lncRNA:CR32773
Fife
unc-13-4A
Eip63F-1
Con
robo2
Cbp53E
dpr3
CG7135
santa-maria
Obp19d
0
1
2
3
4
5
6
7
8
9
10
AST
12
ALPN
15
16
17
C2
20
C3
22
23
24
25
Dm1/Dm10/Dm12
Dm3
Dm9
DOP
30
KC-a/b
KC-a'/b'
34
KC-G
36
37
38
L1/L2/L3
CTX
L4
Lawf1
Lawf2
44
ENS
46
LC14
48
LC16
50
LC4
LC6
53
HE
55
CHI
LLPC1
LPLC1
59
LPLC2
61
Mi15
63
Mt1
OPN
ORN
67
PB/FS
SUB
PEP
PRN
75
76
77
79
Pm1
82
Pm2
Pm3
85
86
PR
SER
T1
91
PL
T2a
T3
98
T4-5a/b
T4-5c/d
Tm1
Tm2/Tm9
Tm20
Tm3
Tm5ab
TmY3
TmY4
TmY5a
111
TmY8
113
Expression Level
8
6
4
2
0
% Expressing
0
25
50
75
100

Figure S1 Single-cell transcriptomic clustering and top 5 gene markers. Gene expression patterns of top 5 marker genes of each cell cluster are shown across all cell clusters (Materials and Methods). The expression level and the percentage of cells in each cell cluster with the gene detected were shown.

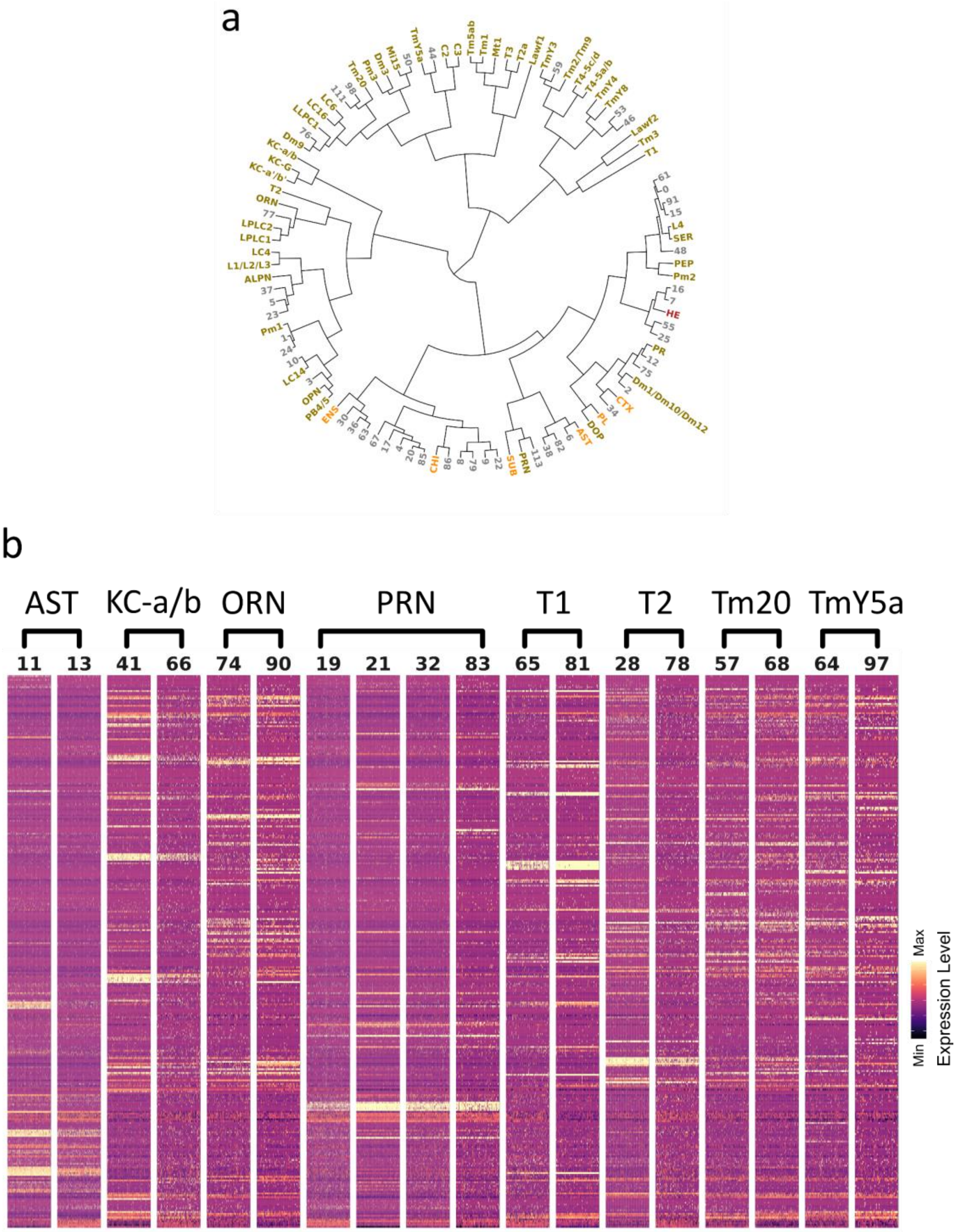

Figure S2 Single-cell transcriptomic clustering.
(a) Tree-shaped dendrogram of the hierarchical relationships among all clusters.
(b) Heatmap showing expression of top 10 cluster marker genes among merged clusters. Merged clusters had similar gene expression patterns.

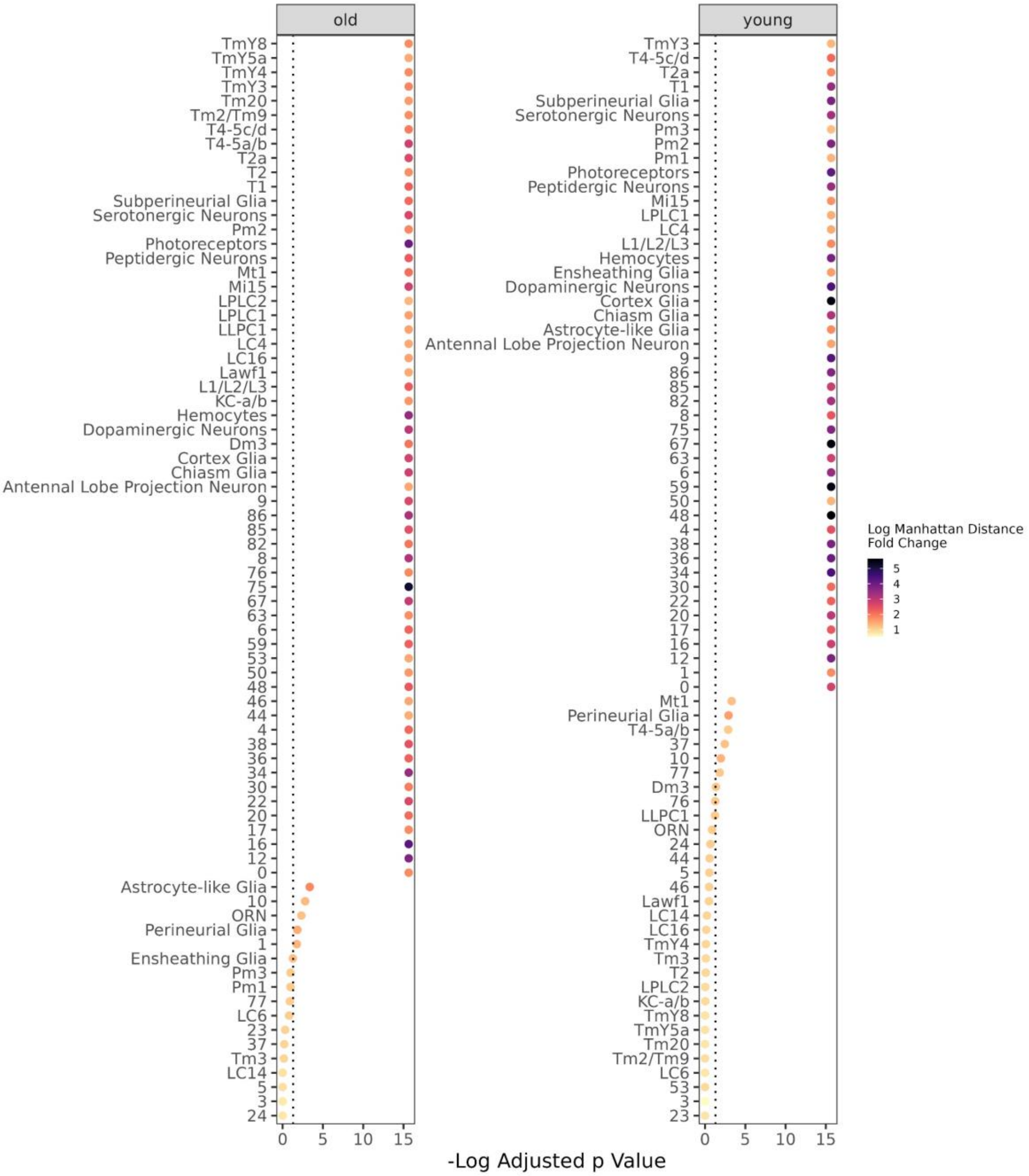

Figure S3 Statistical Significance of Global Transcriptome Shifts. Global transcriptome changes were quantified using the fold change in Manhattan distance across all genes between conventional and axenic fly brains, analyzed separately for young and old groups within each cell cluster. Significance was assessed by performing 20,000 permutations to evaluate the impact of the gut microbiome on each cell cluster in both age groups (Materials and Methods). The vertical dashed line represents the significance threshold ($p < 0.05$).

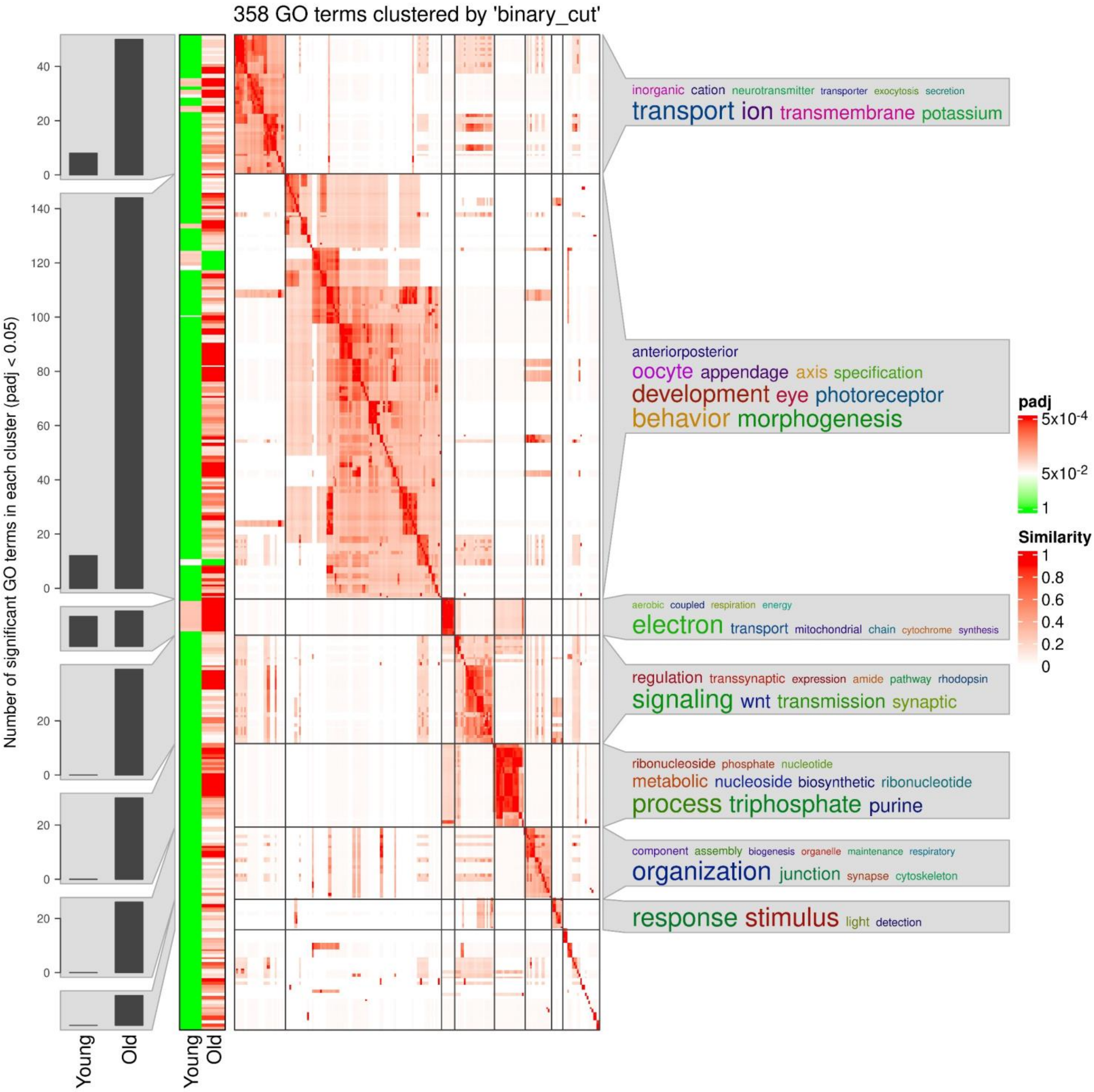

Figure S4. GO enrichment analysis using binary cut clustering between the two different age groups.

A binary cut method was used to cluster the GO terms enriched into groups sharing similar functions. The word cloud labels the information of shared terms in each cluster. The frequency of each term in young and old samples was shown on the left.

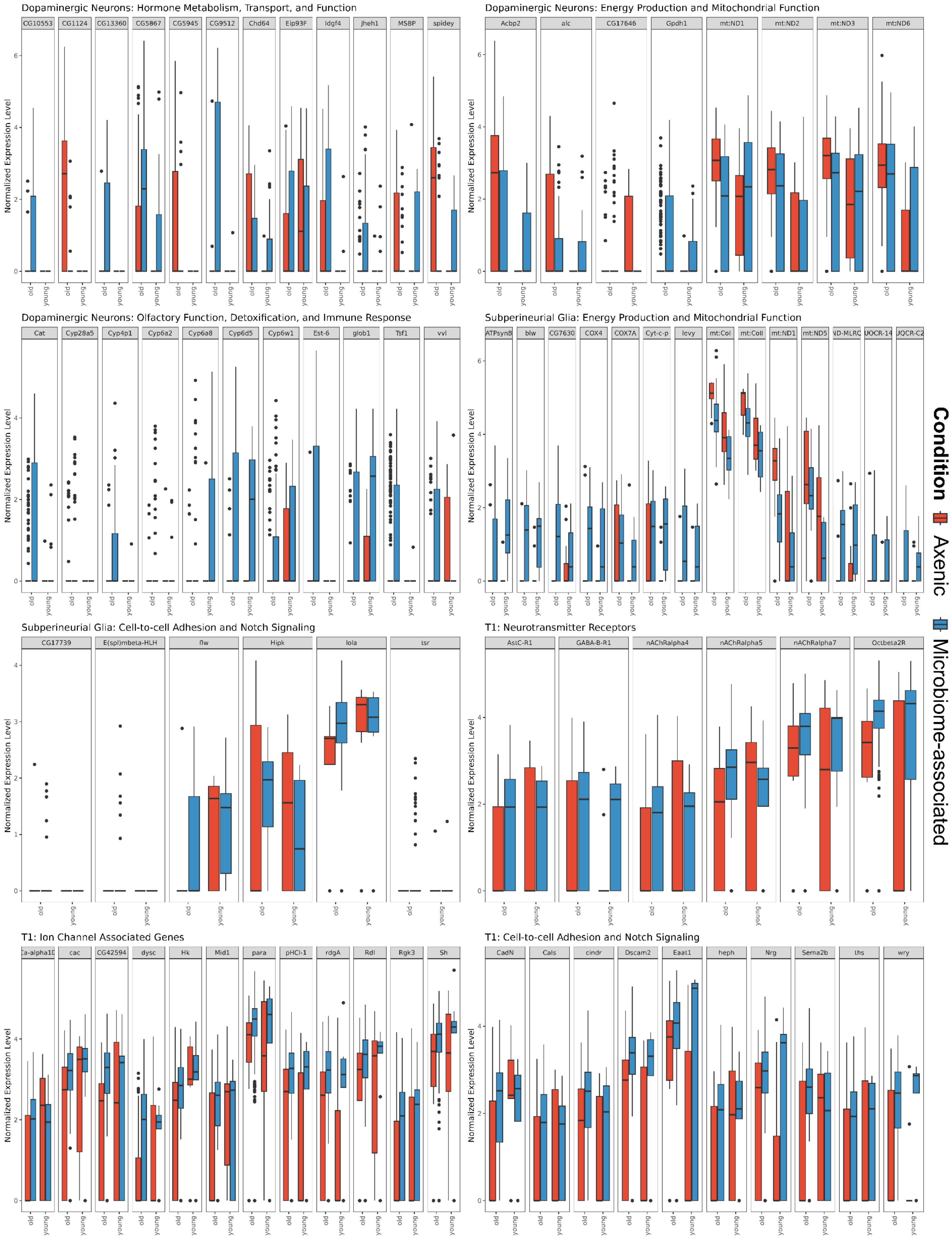
Dopaminergic Neurons: Hormone Metabolism, Transport, and Function
CG10553 CG1124 CG13360 CG5867 CG5945 CG9512 Chd64 Eip93F Idgf4 Jheh1 MSBP spidey
Dopaminergic Neurons: Energy Production and Mitochondrial Function
Acbp2 alc CG17646 Gpdh1 mt:ND1 mt:ND2 mt:ND3 mt:ND6
Dopaminergic Neurons: Olfactory Function, Detoxification, and Immune Response
Cat Cyp28a5 Cyp4p1 Cyp6a2 Cyp6a8 Cyp6d5 Cyp6w1 Est-6 glob1 Tsf1 vvl
Subperineurial Glia: Energy Production and Mitochondrial Function
blw CG7630 COX4 COX7A Cyt-c-p levy mt:CoI mt:CoII mt:ND1 mt:ND5
Subperineurial Glia: Cell-to-cell Adhesion and Notch Signaling
CG17739 E(spl)mbeta-HLH fw Hipk lola tsr
T1: Neurotransmitter Receptors
AstC-R1 GABA-B-R1 nAChRalpha4 nAChRalpha5 nAChRalpha7 Octbeta2R
T1: Ion Channel Associated Genes
cac CG42594 dysc Hk Mid1 para pHCl-1 rdgA Rdl Rgk3 Sh
T1: Cell-to-cell Adhesion and Notch Signaling
CadN Cals cindr Dscam2 Eaat1 heph Nrg Sema2b ths wry
Normalized Expression Level
old
young
Condition
Axenic
Microbiome-associated

Figure S5. Gene expression profile of selected DEGs.
Shown are the expression patterns of top DEGs identified in dopaminergic neurons, subperineurial glia, and T1 neurons across different treatment groups. Genes are grouped by their associated biological functions to highlight functional distinctions in transcriptional responses between cell types and conditions.

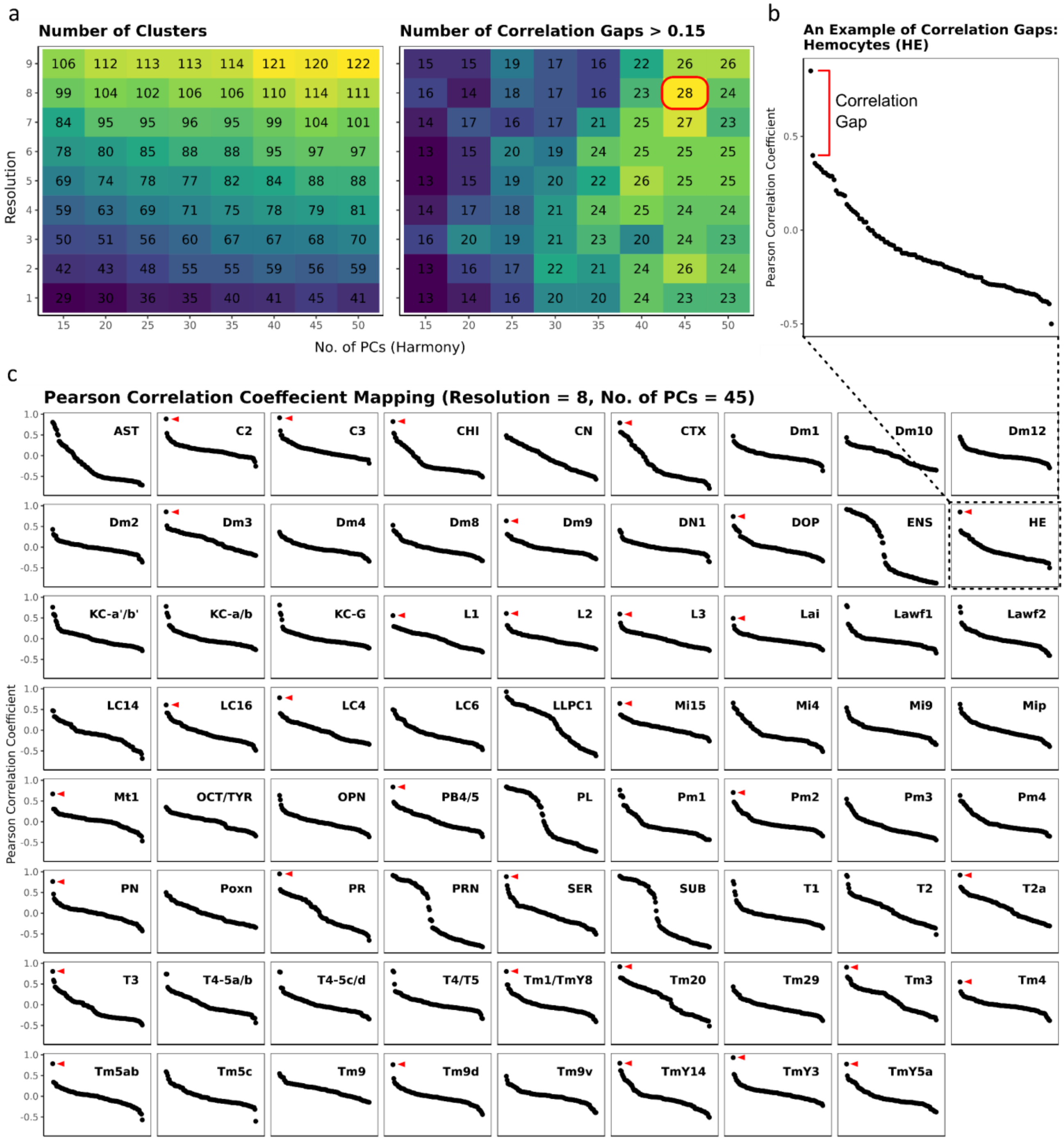

Figure S6 Clustering optimization and biological relevance of clusters. (a) (Left) Number of clusters resulting from different combinations of cluster parameters; (Right) Number of unique reference cell type transcriptomes matching our cluster marker gene profiles, based on a minim gap of Pearson correlation coefficient of 0.15 from various cluster parameter combintations. (b) Pearson correlation coefficient between cluster marker genes mapping to published hemocyte marker genes. (c) Pearson correlation coefficient between cluster marker genes mapping to marker genes of 71 different published cell types.

Supplementary Data S1 Gene Expression Summary by Cell Cluster from scRNA-seq Analysis

Supplementary Data S2 GO-GSEA of DEGs Based on ΠC Index

Supplementary Data S3 Reference Marker Gene Expression Pattern of Known Cell Types
